# Supplementary figures and images for: Interplay Between Stress and Reproduction: Novel Epigenetic Markers in Response to Shearing Patterns in Australian Merino Sheep (Ovis aries)
Source: Front Vet Sci. 2022 Apr 7;9:830450. doi: 10.3389/fvets.2022.830450 (PMC9021797; doi:10.3389/fvets.2022.830450)

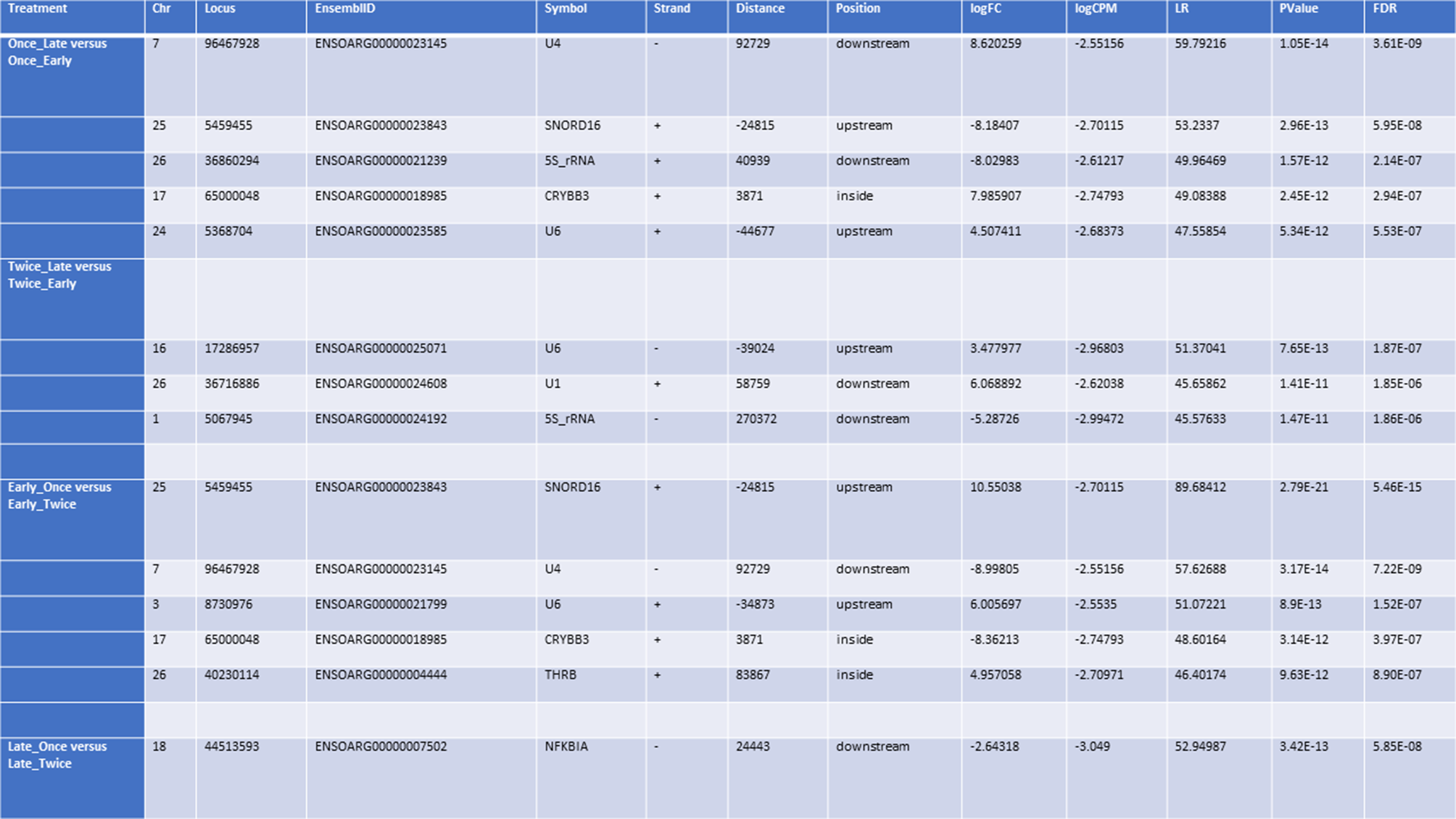

Supplement: Supplementary Table 1 — Annotated epigenetically modulated genes for Merino ewes as determined using gene cards. [file Image_1.tif]

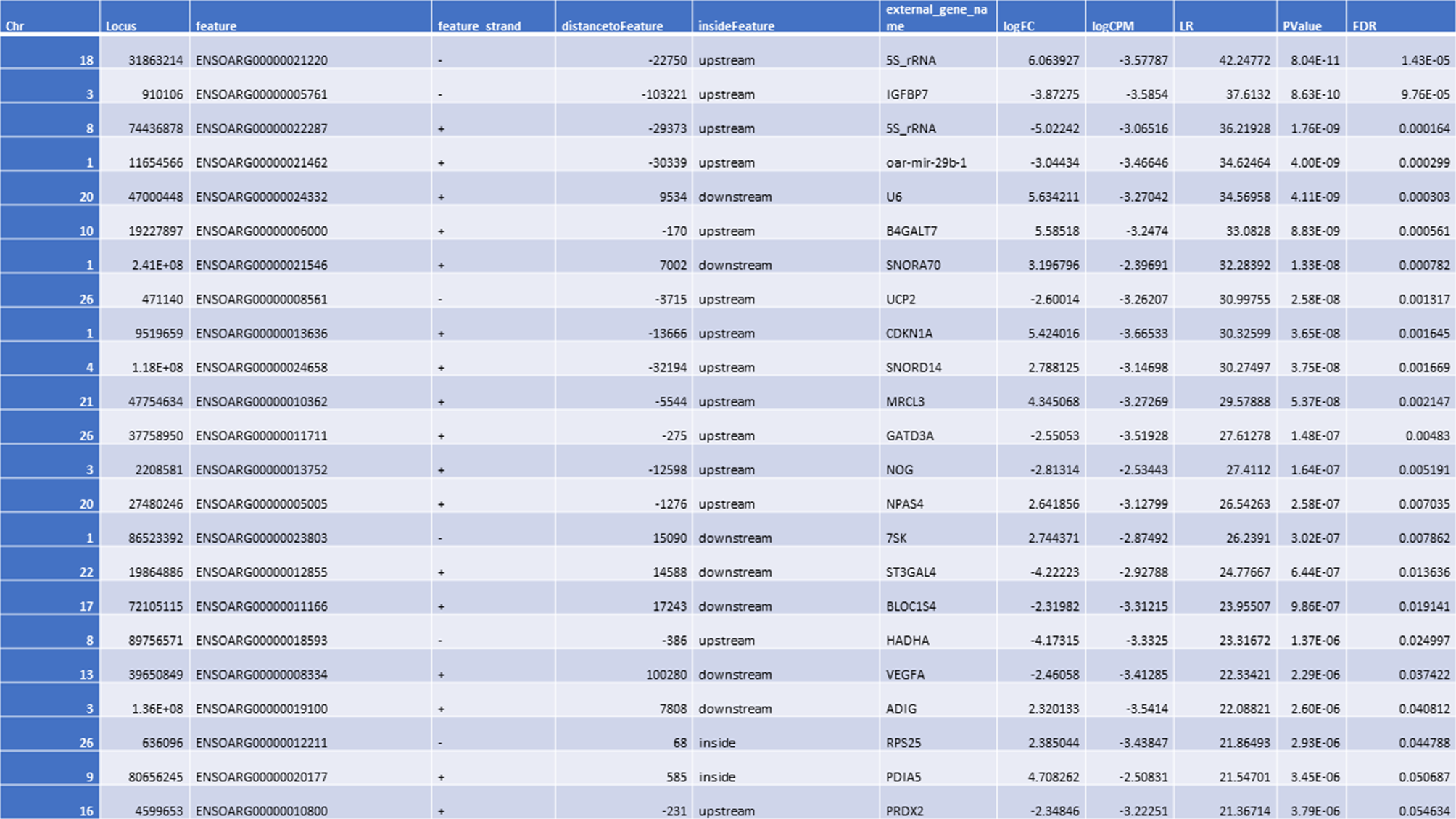

Supplement: Supplementary Table 2 — Annotated epigenetically modulated genes for Merino lambs as determined using gene cards. [file Image_2.tif]
